# Achieving Uniform Li Plating/Stripping at Ultrahigh Currents and Capacities by Optimizing 3D Nucleation Sites and Li_2_Se‐Enriched SEI

**DOI:** 10.1002/advs.202104689

**Published:** 2022-01-24

**Authors:** Jiaqi Cao, Yonghui Xie, Yang Yang, Xinghui Wang, Wangyang Li, Qiaoli Zhang, Shun Ma, Shuying Cheng, Bingan Lu

**Affiliations:** ^1^ College of Physics and Information Engineering Institute of Micro‐Nano Devices and Solar Cells Fuzhou University Fuzhou 350108 China; ^2^ Fujian Science & Technology Innovation Laboratory for Optoelectronic Information of China Fuzhou 350108 China; ^3^ Jiangsu Collaborative Innovation Center of Photovolatic Science and Engineering Changzhou 213164 China; ^4^ College of Materials Science and Engineering Fuzhou University Fuzhou 350108 China; ^5^ School of Physics and Electronics State Key Laboratory of Advanced Design and Manufacturing for Vehicle Body Hunan University Changsha Hunan 410082 China

**Keywords:** flexible lithium metal anodes, lithium F=nucleation sites, lithium metal battery, MOF, SEI

## Abstract

Lithium (Li) has garnered considerable attention as an alternative anodes of next‐generation high‐performance batteries owing to its prominent theoretical specific capacity. However, the commercialization of Li metal anodes (LMAs) is significantly compromised by non‐uniform Li deposition and inferior electrolyte–anode interfaces, particularly at high currents and capacities. Herein, a hierarchical three‐dimentional structure with CoSe_2_‐nanoparticle‐anchored nitrogen‐doped carbon nanoflake arrays is developed on a carbon fiber cloth (CoSe_2_–NC@CFC) to regulate the Li nucleation/plating process and stabilize the electrolyte–anode interface. Owing to the enhanced lithiophilicity endowed by CoSe_2_–NC, in situ‐formed Li_2_Se and Co nanoparticles during initial Li nucleation, and large void space, CoSe_2_–NC@CFC can induce homogeneous Li nucleation/plating, optimize the solid electrolyte interface, and mitigate volume change. Consequently, the CoSe_2_–NC@CFC can accommodate Li with a high areal capacity of up to 40 mAh cm^–2^. Moreover, the Li/CoSe_2_–NC@CFC anodes possess outstanding cycling stability and lifespan in symmetric cells, particularly under ultrahigh currents and capacities (1600 h at 10 mA cm^−2^/10 mAh cm^−2^ and 5 mA cm^−2^/20 mAh cm^−2^). The Li/CoSe_2_–NC@CFC//LiFePO_4_ full cell delivers impressive long‐term performance and favorable flexibility. The developed CoSe_2_–NC@CFC provides insights into the development of advanced Li hosts for flexible and stable LMAs.

## Graphical abstract

A hierarchical CoSe_2_‐nanoparticle‐anchored nitrogen‐doped carbon nanoflake arrays is developed on a carbon fiber cloth for a flexible and stable Li host with excellent cycling performance under ultrahigh currents and capacities.

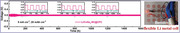

## 1 Introduction

In recent decades, investigations into high‐energy‐density secondary batteries have continued to increase to satisfy the increasing demand for electric vehicles, mobile devices, and flexible electronics.^[^ ^1^, ^2^, ^3^, ^4^ ^]^ Lithium‐ion batteries (LIBs) are ideal candidates owing to their long lifespans and high safety.^[^ ^5^, ^6^ ^]^ However, the development of high‐energy‐density LIBs is limited significantly by the low theoretical capacity of graphite‐based anodes (372 mAh g^–1^).^[^ ^7^, ^8^ ^]^ Among the available anode materials, lithium (Li) metal is considered a highly demanded electrode material owing to its high theoretical capacity (3860 mAh g^–1^) and lowest electrochemical potential (−3.04 V vs the standard hydrogen electrode), which enable high‐energy densities to be realized in full‐cell configurations.^[^ ^9^, ^10^, ^11^, ^12^ ^]^ However, despite its notable merits, the commercialization of Li metal anodes (LMAs) is impeded by its inherent drawbacks, as follows: 1) A continuous side reaction stemming from a fragile solid electrolyte interface (SEI) results in the accumulation of “dead Li”; 2) infinite volume change during the Li plating/stripping process causes the destruction of the electrode structure and an unstable electrolyte/electrode interface; 3) inhomogeneous Li nucleation incurs unrestrained Li dendrite growth, which can result in short circuits, fires, and explosions.^[^ ^13^, ^14^, ^15^, ^16^ ^]^

Significant efforts have been expended to address the abovementioned issue. One strategy is to reinforce the stability of the electrolyte–electrode interface to suppress the formation of Li dendrites. For example, the utilization of novel electrolyte additives, such as fluoroethylene carbonate, LiNO_3_, and Li_2_S_8_, has been verified to improve SEI strength and promote uniform Li deposition.^[^ ^17^, ^18^, ^19^ ^]^ In addition, physical constraints, including the design of a robust artificial protective layer or solid electrolyte, can effectively inhibit dendrite growth.^[^ ^20^, ^21^, ^22^, ^23^ ^]^ However, owing to the “hostless” nature of LMAs, the abovementioned strengthened interfaces have limited space for accommodating Li deposition, particularly in long‐term cycles.^[^ ^24^ ^]^ The repeated volume fluctuation inevitably damages the SEI and exposes fresh Li to the electrolyte, causing an increase in the internal impedance and dendrite growth, and eventually the failure of the battery.^[^ ^25^ ^]^ In this regard, volume change during the cycle must be alleviated to achieve high‐performance LMAs.

More recently, the use of three‐dimentional (3D) conductive frameworks to accommodate Li plating/stripping has achieved remarkable results.^[^ ^26^, ^27^, ^28^ ^]^ Owing to their light mass, high conductivity, predominant flexibility, and robust structure, carbon‐based frameworks are promising high‐energy‐density Li hosts among numerous frameworks, as well as in the area of flexible energy storage.^[^ ^29^, ^30^ ^]^ The introduction of lithiophilic materials into carbon‐based frameworks is a representative strategy for promoting Li storage performance.^[^ ^31^, ^32^ ^]^ The enhanced lithiophilicity of Li reduces the Li nucleation barrier, which can induce uniform Li nuclei and eradicate dendrite formation. For example, Liu et al. designed an MgO nanosheet‐anchored carbon fiber cloth as a Li host with a prolonged lifespan exceeding 500 h at 1 mA cm^–2^/1 mAh cm^–2^.^[^ ^33^ ^]^ Zhou et al. prepared a lithiophilic CC@CN–Co framework to accommodate Li that can be cycled for 1000 h at 5 mA cm^−2^/5 mAh cm^–2^.^[^ ^34^ ^]^ Although it exhibits favorable cycling stability by homogenizing the Li nucleation/plating behavior, its current and capacities are insufficient for satisfying the requirements of practical applications; this is attributable to their unstable SEI. Hence, it is highly desirable to investigate a novel carbon‐based framework with lithiophilic sites and an optimized SEI to realize homogeneous Li nucleation and robust electrolyte–anode interfaces for flexible and stable energy‐storage devices with high capacities.

In this study, we developed 3D hierarchical CoSe_2_‐nanoparticle‐anchored nitrogen‐doped carbon (CoSe_2_–NC) nanoflake arrays on a carbon fiber cloth (CoSe_2_–NC@CFC) as a flexible Li host for dendrite‐free LMAs with high capacities. In this novel design, the CoSe_2_–NC@CFC offers the following advantages: first, the uniform distribution of doped N‐bonding configurations and CoSe_2_ nanoparticles endows CFC with excellent lithiophilicity, thereby facilitating uniform Li deposition behavior with a small overpotential. Second, owing to the high reducibility of Li, CoSe_2_ nanoparticles are converted into Li_2_Se/Co nanoparticles during the initial Li nucleation process, which can serve as ordered Li nucleation sites to regulate Li deposition. More importantly, the in situ‐generated Li_2_Se‐enriched SEI possesses high ionic conductivity, chemical stability, and mechanical strength, which are favorable for rapid Li^+^ transport and electrolyte–anode interface stability.^[^ ^35^, ^36^ ^]^ Additionally, the enlarged specific surface area and porous structure of the CoSe_2_–NC@CFC significantly decrease the local current density and minimize volume expansion, thereby guaranteeing steady cycling behavior. Consequently, the CoSe_2_–NC@CFC allows the prestoring of Li at an ultrahigh areal capacity of up to 40 mAh cm^–2^ (Li/CoSe_2_–NC@CFC). In addition, the as‐obtained Li/CoSe_2_–NC@CFC exhibits an impressive lifespan exceeding 1600 h with high areal capacities of 10 and 20 mAh cm^–2^ at 10 and 5 mA cm^–2^, respectively. More importantly, when coupled with LiFePO_4_ (LFP), the Li/CoSe_2_–NC@CFC//LFP exhibits an excellent capacity retention of 86.4% after 1000 cycles at a high rate of 2 C and is a promising material for flexible LMAs. Our study provides insights into fabrication strategies for advanced high‐energy‐density LMAs.

## 2 Results and Discussion

### 2.1 Preparation and Characterization of CoSe_2_–NC@CFC

**Figure 1:**
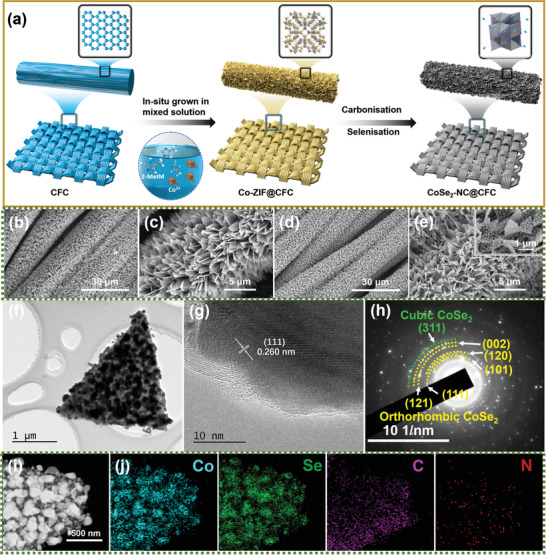
a) Schematic diagram of preparation route for CoSe_2_–NC@CFC. SEM images of b,c) Co‐ZIF@CFC and d,e) CoSe_2_–NC@CFC. Inset in (e) shows high‐magnification SEM image of CoSe_2_–NC@CFC. f) TEM, g) HRTEM, h) SAED, i) HAADF–STEM, and j) elemental mapping images of CoSe_2_–NC nanoflake.

A schematic illustration of the preparation route for the CoSe_2_–NC@CFC is presented in **Figure** **1**a. First, Co‐based zeolitic imidazolate framework (Co‐ZIF) nanoflake arrays were decorated in situ on a CFC (Co‐ZIF@CFC) via room‐temperature crystallization in a mixed solution of 2‐methylimidazole and Co(NO_3_)_2_·6H_2_O.^[^ ^37^ ^]^ As shown in Figures 1b,c, compared with the smooth surface of the pristine CFC (Figure S1, Supporting Information), the Co‐ZIF@CFC showed evenly distributed dense nanoflake arrays. Subsequently, after high‐temperature carbonization and selenization, the blue Co‐ZIF@CFC was converted into black CoSe_2_–NC@CFC (Figure S2, Supporting Information), which maintained the nanoflake‐like morphology and flexibility (Figure 1d,e; Figure S3, Supporting Information). In addition, numerous nanoparticles were anchored in the carbon nanoflake arrays, different from the Co‐ZIF@CFC. More importantly, the hierarchical CoSe_2_–NC@CFC had a large active surface area (21.62 m^2^ g^–1^) and numerous pores (pore size: 4.15 nm; Figure S4, Supporting Information), which can effectively reduce the local current density and provide sufficient space to maintain the volume stability.^[^ ^33^ ^]^ Transmission electron microscopy (TEM) was employed to visualize the microstructures of CoSe_2_–NC. Figure 1f shows that the nanoflakes had a triangular shape, and that the diameter of the embedded nanoparticles was ≈200 nm. High‐resolution TEM (HRTEM) image of CoSe_2_–NC in Figure 1g shows the lattice fringes of the orthorhombic CoSe_2_ (111) plane with a plane spacing of 0.260 nm.^[^ ^38^ ^]^ In particular, the selected area electron diffraction (SAED) pattern (Figure 1h) shows that the CoSe_2_ nanoparticles exhibited orthorhombic (o‐CoSe_2_) and cubic (c‐CoSe_2_) phases. As shown in Figure 1i,j, the HAADF–STEM and elemental mapping images of the CoSe_2_–NC nanoflake show that the spatial distributions of the nanoparticles matched well with those of Co and Se, confirming that the nanoparticles were composed of CoSe_2_. Additionally, the uniformly distributed C and N revealed successful N doping in the carbon nanoflakes. Moreover, the scanning electron microscopy (SEM) elemental mapping images and corresponding energy‐dispersive X‐ray spectroscopy (EDX) analysis of the CoSe_2_–NC@CFC were consistent with the conclusions above, i.e., the atomic ratio of Co to Se was ≈1:2, whereas N constituted 2.19% of the CoSe_2_–NC@CFC sample (Figures S5 and S6, Supporting Information).

**Figure 2:**
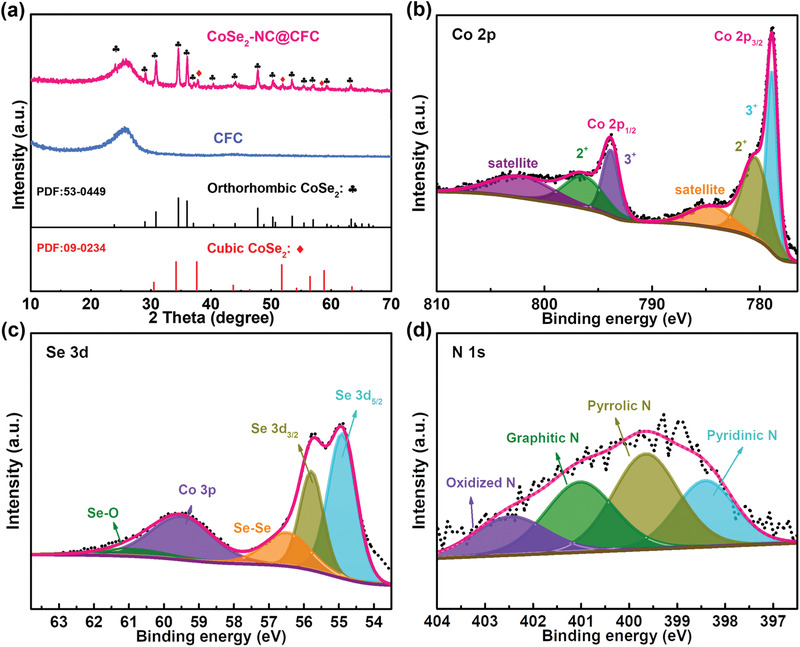
a) XRD patterns of CFC and CoSe_2_–NC@CFC. XPS spectra of CoSe_2_–NC@CFC: b) Co 2p, c) Se 3d, and d) N 1s.

X‐ray diffraction (XRD) patterns of the CFC, Co‐ZIF@CFC, and CoSe_2_–NC@CFC were characterized, as shown in **Figure** **2**a and Figure S7 (Supporting Information). The diffraction peaks of the CoSe_2_–NC@CFC can be assigned to o‐CoSe_2_ (PDF#43‐0449) and c‐CoSe_2_ (PDF#09‐0234), separately.^[^ ^39^ ^]^ The same results were obtained from the XRD and SAED patterns, in that the nanoparticles were primarily composed of o‐CoSe_2_ with a slight amount of c‐CoSe_2_. X‐ray photoelectron spectroscopy (XPS) analysis was performed to investigate the chemical bonding property of the CoSe_2_–NC@CFC. A full XPS of the CoSe_2_–NC@CFC (Figure S8, Supporting Information), Co, Se, N, C, and O was performed. As shown in Figure 2b, the two deconvoluted peaks of the Co 2p spectrum at 780.4 and 796.3 eV belonged to Co 2p_3/2_ and Co 2p_1/2_, respectively, corresponding to Co^2+^ in CoSe_2_.^[^ ^40^, ^41^ ^]^ Additionally, the presence of the Co^3+^ peak was ascribed to the partial surface oxidation of Co.^[^ ^42^ ^]^ the XPS spectrum of Se 3d is shown in Figure 2c. The peaks at 54.9 and 55.8 eV corresponded to Se 3d_5/2_ and Se 3d_3/2_, respectively, which coincided with Co–Se bonding in CoSe_2_.^[^ ^43^ ^]^ The peak at 56.5 eV was attributed to a trace amount of metalloid Se remaining during selenization.^[^ ^44^ ^]^ Moreover, the peaks located at 58–62 eV were associated with Co 3p and Se–O bonding.^[^ ^42^ ^]^ The N 1s XPS spectrum was composed of four peaks at 398.4, 399.7, 401.0, and 402.5 eV, which corresponded to pyridinic N, pyrrolic N, graphitic N, and oxidized N, respectively (Figure 2d).^[^ ^45^ ^]^ Existing N‐containing functional groups can improve the conductivity and lithiophilicity of the electrode, thereby substantially decreasing the Li nucleation barrier and promoting a smooth Li deposition.^[^ ^32^, ^46^ ^]^

### 2.2 Modification mechanism and morphology evolution of CoSe_2_–NC@CFC

**Figure 3:**
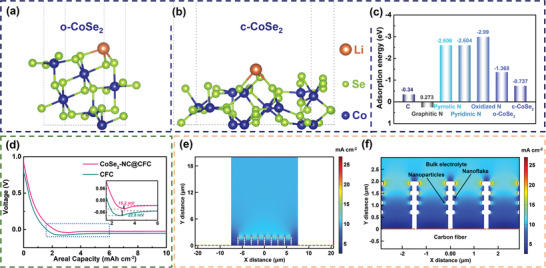
DFT calculations of Li atom with a) o‐CoSe_2_ and b) c‐CoSe_2_. c) Comparison of E_ad_ of one Li atom adsorbed on C, graphitic N, pyrrolic N, pyridinic N, oxidized N, o‐CoSe_2_, and c‐CoSe_2_. d) Voltage profiles of CoSe_2_–NC@CFC and CFC during initial Li deposition at 1 mA cm^–2^. e) FEA of current density distribution at nanoflakes–electrolyte interface and f) its partially enlarged figure.

To interpret the lithiophilic improvement of CoSe_2_–NC@CFC comprehensively, density functional theory (DFT) calculations were performed to compare the adsorption energy (E_ad_) of one Li atom with carbon, N‐containing functional groups, and CoSe_2_ (**Figure** **3**a–c; Figure S9, Supporting Information). Based on the calculated results shown in Figure 3c, the E_ad_ values of pyrrolic N (−2.606 eV), pyridinic N (−2.604 eV), oxidized N (−2.99 eV), o‐CoSe_2_ (−1.368 eV), and c‐CoSe_2_ (−0.737 eV) were relatively higher than those of defect‐free carbon (−0.34 eV) and graphitic N (0.273 eV). Briefly, the higher the adsorption energy, the stronger the interaction, and the lower the nucleation barrier with Li atoms. Hence, in contrast to the CFC (Figure 3d), the presence of these sites with a higher E_ad_ improves the lithiophilicity of the entire electrode, which can significantly reduce the Li nucleation overpotential of the CoSe_2_–NC@CFC (15.2 mV, i.e., 50.7% lower than that of the CFC), resulting in homogeneous Li plating.

An XRD analysis was conducted to investigate the reaction products of CoSe_2_ and Li. The tested sample was CoSe_2_–NC@CFC after electroplating 10 mAh cm^–2^ Li at 1 mA cm^–2^. As shown in Figure S10 (Supporting Information), the emergence of new peaks replaced the characteristic peaks of CoSe_2_, as shown in Figure 2a. The diffraction peaks at 25.9°, 30.0°, 42.9°, and 50.7° corresponded to the (111), (200), (220), and (311) crystal planes of Li_2_Se, respectively, whereas the diffraction peak at 43.9° corresponded to metallic Co. In addition, the strong diffraction peaks at 36.5° and 52.3° reflect the deposition of metallic Li, whereas the weak peak at 24.1° indicates the formation of lithiophilic LiC_6_ via Li^+^ intercalation, which facilitated Li deposition.^[^ ^47^, ^48^ ^]^ Therefore, the XRD results suggest that the CoSe_2_ nanoparticles would react with Li^+^ during the initial Li nucleation and transform it into Co nanoparticles and Li_2_Se. This process can be expressed as follows:

(1) $$\mathrm{CoS}e_{2}+4\mathrm{Li}\to \mathrm{Co}+2Li_{2}\mathrm{Se}$$

Notably, the in situ‐formed Li_2_Se possessed high ionic conductivity, mechanical resistance, and chemical stability, which prevented uncontrollable dendrite growth and ensured the stability of the electrolyte/anode interface, thereby yielding an ideal SEI.^[^ ^35^, ^36^ ^]^ Moreover, the in situ‐formed Li_2_Se/Co nanoparticles were conducive to regulating the currents evenly and realizing uniform Li deposition, as demonstrated via simulations based on the finite elemental analysis (FEA) method. The constructed module is shown in Figure S11 (Supporting Information), and the entire area can be classified into three regions: 1) Y > 2 µm represents the bulk electrolyte; 2) 0 < Y < 2 µm represents the vertical N‐doped carbon nanoflakes with embedded Li_2_Se/Co nanoparticles; 3) Y < 0 µm represents the carbon fiber. As shown in Figure 3e,f, the current density at the Li_2_Se/Co nanoparticles is significantly higher than that in other areas. This indicates that the Li_2_Se/Co nanoparticles can induce Li^+^ aggregation and serve as Li nucleation sites. More importantly, because of the uniform distribution of the Li_2_Se/Co nanoparticles on the N‐doped carbon nanoflakes, the Li nucleation on the entire nanoflake tends to be uniform, which promotes smooth Li plating in the subsequent deposition and prevents the formation of irregular Li dendrites.

**Figure 4:**
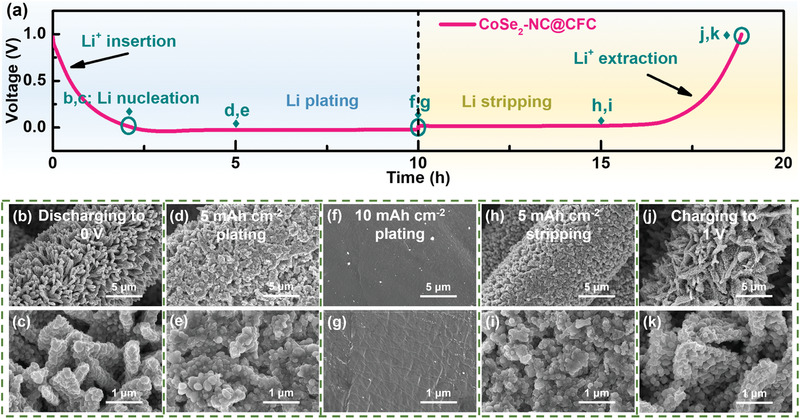
a) Voltage profile of Li plating/stripping process on CoSe_2_–NC@CFC at 1 mA cm^–2^ with capacity of 10 mAh cm^–2^. SEM images of CoSe_2_–NC@CFC morphology evolution: b,c) discharging to 0 V, plating Li of d,e) 5 mAh cm^–2^ and f,g) 10 mAh cm^–2^; h,i) stripping Li of 5 mAh cm^–2^ and j,k) charging to 1 V.

To confirm the critical role of the hierarchical CoSe_2_–NC nanoflake arrays in promoting the homogeneous deposition of Li, the morphology evolution of the CoSe_2_–NC@CFC was investigated during galvanostatic charge–discharge processes at 1 mA cm^–2^ with a capacity of 10 mAh cm^–2^. As shown in **Figure** **4**a, the Li plating/stripping process on CoSe2–NC@CFC comprised five stages: Li^+^ insertion, Li nucleation, Li plating, Li stripping, and Li^+^ extraction. When the voltage discharged to 0 V, compared with the pristine morphology of the CoSe_2_–NC nanoflake arrays (Figure 1d,e), the N‐doped carbon nanoflakes and nanoparticles became thicker owing to the insertion of Li^+^ and the reduction of CoSe_2_, followed by the in situ formation of Co nanoparticles, a Li_2_Se‐enriched SEI, and lithiophilic LiC_6_ (Figure 4b,c). As shown by the FEA results shown in Figure 3f,g, along with a continuous discharge, Li^+^ began to nucleate on the Li_2_Se/Co nanoparticles. After discharging to 5 mAh cm^–2^, the deposited Li on each nanoparticle was fused, and the nanoflakes were gradually filled with Li, which shows that the Li deposition transferred from the nanoparticles to the entire surface of the nanoflakes (Figure 4d,e). When the deposition capacity reached 10 mAh cm^–2^, the surface of the CoSe_2_–NC@CFC was enveloped by a flat Li layer, confirming that the CoSe_2_–NC nanoflake arrays can inhibit Li dendrite growth (Figure 4f,g). By contrast, many irregular Li dendrites grew profusely on the surface of the bare CFC after plating 10 mAh cm^–2^ of Li (Figure S12a, Supporting Information), owing to its lithiophobic nature. In addition, the CoSe_2_–NC@CFC successfully maintained the volume (Figure S13, Supporting Information). It is noteworthy that the Li loading capacity of CoSe_2_–NC@CFC can be further increased to 40 mAh cm^–2^ with a dendrite‐free morphology and steady volume (≈326 µm), as metallic Li is stored completely within the 3D framework instead of accumulating extensively on the surface (Figure S14a–d, Supporting Information). The subsequent process involved Li stripping. After 5 mAh cm^–2^ of Li stripping, the carbon fibers were re‐exposed and partial nanoparticles were observed (Figure 4h,i). Finally, when the voltage was charged to 1 V (Figure 4j,k), the CoSe_2_–NC@CFC remained intact, demonstrating its outstanding reversibility for Li plating/stripping, whereas the bare CFC yielded residual Li (Figure S12b, Supporting Information). DFT calculations, FEA simulations, and experiments suggest that the Li/CoSe_2_–NC@CFC anode offers many structural and compositional advantages toward the development of high‐performance LMAs.

### 2.3 Electrochemical performance

**Figure 5:**
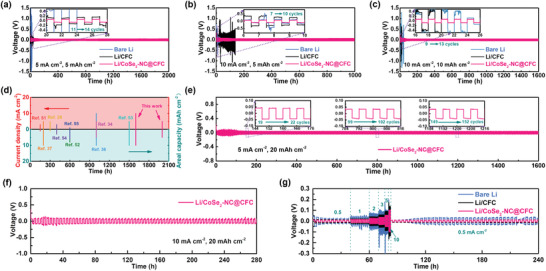
Electrochemical performance of symmetric cells. Galvanostatic cycling of bare Li, Li/CFC, and Li/CoSe_2_–NC@CFC anodes at a) 5 mA cm^–2^/5 mAh cm^–2^, b) 10 mA cm^–2^/5 mAh cm^–2^, and c) 10 mA cm^–2^/10 mAh cm^–2^. d) Comparison of cycling lifespan of Li/CoSe_2_–NC@CFC anode with various reported LMAs in symmetric cells. Galvanostatic cycling of Li/CoSe_2_–NC@CFC anodes at e) 5 mA cm^–2^/20 mAh cm^–2^ and f) 10 mA cm^–2^/20 mAh cm^–2^. g) Rate performances of bare Li, Li/CFC, and Li/CoSe_2_–NC@CFC anodes. Insets show detailed voltage profiles of Li stripping/plating process.

Symmetric cells were assembled to determine the long‐term cycling performance of the Li/CoSe_2_–NC@CFC. As shown in Figure S15 (Supporting Information), the initial overpotentials of the symmetric cells based on bare Li, Li/CFC, and Li/CoSe_2_–NC@CFC were similar at 1 mA cm^–2^/1 mAh cm^–2^. However, the overpotentials of the bare Li and Li/CFC started to fluctuate or increase significantly at ≈100 and 160 h, respectively. This is primarily due to the increase in internal resistance caused by inhomogeneous Li deposition and the accumulation of “dead Li” on the bare Li and Li/CFC anodes, as shown by the SEM results in Figure S16 (Supporting Information). Furthermore, the Li/CoSe_2_–NC@CFC prolonged the cycling life to 900 h. When the cycling capacity and current density increased to 5 mAh cm^–2^/5 mA cm^–2^, the symmetric Li/CoSe_2_–NC@CFC cell (**Figure** **5**a) demonstrated stable Li stripping/plating behaviors and an impressive cycling life exceeding 2000 h (1000 cycles). By contrast, the symmetric cells of the bare Li and Li/CFC can only cycle stably for 6 and 20 h, respectively. Even at an ultrahigh current density of 10 mA cm^–2^ with a capacity of 5 (Figure 5b) or 10 mAh cm^–2^ (Figure 5b,c), the symmetric Li/CoSe_2_–NC@CFC cell maintained a stable voltage platform for 1000 and 1600 h, respectively, whereas the overpotentials of the bare Li and Li/CFC increased or fluctuated rapidly within a few cycles. These results indicate that under high current density and cycling capacity, the bare Li and Li/CFC electrodes could not ensure a uniform Li deposition and a stable electrolyte–anode interface, resulting in an abrupt increase in voltage.^[^ ^49^, ^50^ ^]^ By comparison, as shown in Figure S17 (Supporting Information), the Li/CoSe_2_–NC@CFC did not form Li dendrites after cycling at 10 mA cm^–2^/10 mAh cm^–2^. It is noteworthy that such remarkable cycling performances are superior to those of previously reported LMAs based on 3D frameworks (Figure 5d; Table S1, Supporting Information).^[^ ^28^, ^34^, ^36^, ^37^, ^51^, ^52^, ^53^, ^54^, ^55^ ^]^ To investigate the cycling performance of the Li/CoSe_2_–C@CFC anode under a higher capacity, the executed capacity was further enhanced to 20 mAh cm^–2^. As shown in Figure 5e, at a current density of 5 mA cm^–2^, the symmetric Li/CoSe_2_–NC@CFC cell exhibited a stable voltage platform that remained for over 1600 h. Moreover, the symmetric cell of the Li/CoSe_2_–NC@CFC can be cycled for over 280 h at 10 mA cm^–2^ (Figure 5f), representing its superiority as a high‐energy‐density LMA. In addition, the Coulombic efficiency of the CoSe_2_–NC@CFC//Li half‐cell was 98.7% on average, which was much higher than that of the bare CFC (Figure S18, Supporting Information).

The rate performances of the bare Li, Li/CFC, and Li/CoSe_2_–NC@CFC anodes were further investigated at 0.5, 1, 2, 3, 5, and 10 mA cm^–2^ with a fixed capacity of 1 mAh cm^–2^. As shown in Figure 5g, the overpotentials of the Li/CoSe_2_–NC@CFC were lower than those of the bare Li and Li/CFC, particularly under high current densities. In addition, when the current density returned to 0.5 mA cm^–2^, the overpotential of the Li/CoSe_2_–NC@CFC returned to ≈8 mV, whereas the overpotential of the symmetric cells of the bare Li and Li/CFC increased gradually to 30 and 14 mV, respectively. Such low and stable overpotentials indicate the fast ion/electron transport kinetics and robust electrolyte–anode interface realized at the Li/CoSe_2_–NC@CFC anode, which were further elucidated using electrochemical impedance spectroscopy. Nyquist plots of bare Li, Li/CFC, and Li/CoSe_2_–NC@CFC in symmetric cells and the equivalent circuits are shown in Figures S19 and S20 (Supporting Information). As listed in Table S2 (Supporting Information), the Li/CoSe_2_–NC@CFC exhibited a much lower SEI film resistance (R_SEI_, 3.1 Ω) and charge–transfer resistance (R_CT_, 2.9 Ω) than the bare Li (4.5 and 27.1 Ω, respectively) and Li/CFC (12.1 and 9.4 Ω, respectively) before the cycling, which can be attributed to the formation of a Li_2_Se‐enriched SEI. After five cycles, the R_SEI_ and R_CT_ of the Li/CoSe_2_–NC@CFC decreased slightly to 2.4 and 2.3 Ω, respectively, demonstrating the reduced polarization and optimized interface between the electrolyte and anode.

**Figure 6:**
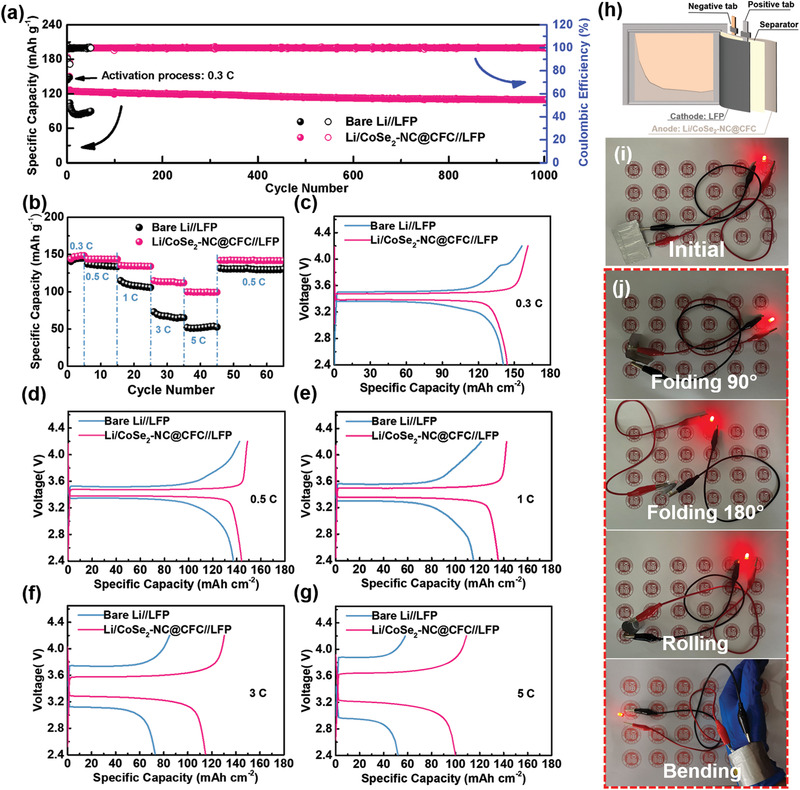
Electrochemical performances of full cells. a) Long‐term cycling performance of Li//LFP and Li/CoSe_2_–NC@CFC//LFP cells at 2 C. b) Rate performance and c–g) voltage profiles of Li//LFP and Li/CoSe_2_–NC@CFC//LFP cells cycled from 0.3 to 5 C. h) Schematic illustration of Li/CoSe_2_–NC@CFC//LFP pouch cell. i,j) Illuminating test of Li/CoSe_2_–NC@CFC//LFP flexible pouch cell under various folded states.

The potential of the Li/CoSe_2_–NC@CFC anode for practical applications was evaluated in full‐cell tests, in which the LiFePO_4_ (LFP) was selected as the cathode paired with bare Li (Li//LFP) or Li/CoSe_2_–NC@CFC (Li/CoSe_2_‐NC@CFC//LFP) anodes. The active mass of LFP was ≈6 mg cm^–2^. As shown in **Figure** **6**a, the long‐term cycling performances of Li//LFP and Li/CoSe_2_‐NC@CFC//LFP cells were scrutinized at 2 C. The Li/CoSe_2_‐NC@CFC//LFP exhibited outstanding cycling stability over 1000 cycles and retained a high capacity of 109.4 mAh g^–1^ with a capacity retention of 86.4%. By contrast, the capacity of Li//LFP decayed rapidly to 83.9 mAh g^–1^ after 20 cycles. Moreover, the Li/CoSe_2_–NC@CFC anode exhibited enhanced rate performance. As shown in Figure 6b, the discharge capacities of the Li/CoSe_2_–NC@CFC//LFP and Li//LFP cells were similar at the initial rate of 0.3 C, i.e., 144.1 and 140.7 mAh g^–1^, respectively. As the rate increased, the Li/CoSe_2_–NC@CFC//LFP cell exhibited high discharge capacities of 144.0, 135.5, 114.8, and 99.8 mAh g^–1^ at 0.5, 1, 3, and 5 C, respectively, whereas those of the Li/LFP cell declined significantly to 137.2, 114.9, 73.0, and 52.0 mAh g^–1^ at 0.5, 1, 3, and 5 C, respectively. This was primarily due to the high polarization of Li//LFP caused by the sluggish charge‐transfer behavior and high internal resistance (Figure 6c–g). The morphological differences between the bare Li and Li/CoSe_2_–NC@CFC anodes after the rate cycles are shown in Figure S21 (Supporting Information). It was observed that the surface of the bare Li contained numerous cracks and fragments, accompanied by the accumulation of Li dendrites and “dead Li,” whereas the Li/CoSe_2_–NC@CFC anode maintained a uniform morphology.

To further demonstrate the feasibility of applying Li/CoSe_2_–NC@CFC in flexible devices, a pouch cell was assembled by stacking the Li/CoSe_2_–NC@CFC anode, separator, and LFP cathode (Figure 6h). As shown in Figure S22 (Supporting Information), the as‐prepared Li/CoSe_2_–NC@CFC anode exhibited excellent flexibility compared with the CoSe_2_–NC@CFC. Moreover, the assembled Li/CoSe2–NC@CFC//LFP pouch cell possessed a high capacity retention of 86.1% for 40 cycles at 0.5 C (Figure S23, Supporting Information). In addition, the pouch cell can power a light‐emitting diode under different folding, rolling, and bending states (Figure 6i,j). Hence, the results above show that the Li/CoSe_2_–NC@CFC anode is promising for developing flexible energy‐storage devices with high capacities.

## 3 Conclusions

In this study, an advanced framework (CoSe_2_–NC@CFC) was developed to serve as a flexible Li host for high‐performance LMAs. Under the synergistic effects of excellent lithiophilicity, a 3D Li_2_Se‐enriched SEI, homogeneous Li nucleation sites, and a porous framework, the hierarchical CoSe_2_–NC@CFC successfully promoted homogeneous Li nucleation/plating, stabilized the electrolyte–anode interface, accommodated volume expansion, and provided fast charge transfer kinetics during Li stripping/plating at high currents and capacities, as indicated by DFT calculations, finite element simulations, and experiments. Notably, the maximum areal capacity of prestored Li in the CoSe_2_–NC@CFC was 40 mAh cm^–2^, without distinct dendrite formation and volume changes. The as‐prepared Li/CoSe_2_–NC@CFC anodes delivered an ultralong lifespan of 1600 h (800 cycles) at an ultrahigh current density of 10 mA cm^–2^ and a cycling capacity of 10 mAh cm^–2^ in the symmetric cell. At a higher capacity of 20 mAh cm^–2^, the Li/CoSe_2_–NC@CFC anodes maintained an ultrastable cycling performance for 1600 h. Furthermore, the assembled full cell demonstrated an impressive long‐term cycling performance for more than 1000 cycles, and the pouch cell functioned as intended under various folded states. The proposed optimization strategy combined with structure and SEI modifications proved to be feasible for the development of flexible and stable LMAs.

## Conflict of Interest

The authors declare no conflict of interest.

## Supporting information

Supporting Information

## Data Availability

The data that support the findings of this study are available from the corresponding author upon reasonable request.
