# Supplementary material for: Achieving Uniform Li Plating/Stripping at Ultrahigh Currents and Capacities by Optimizing 3D Nucleation Sites and Li2Se‐Enriched SEI
Source: Adv Sci (Weinh). 2022 Jan 24;9(9):2104689. doi: 10.1002/advs.202104689 (PMC8948610; doi:10.1002/advs.202104689)
Supplement: Supplementary file 1 — Supporting Information [file ADVS-9-2104689-s001.pdf]

ADVANCED SCIENCE
Open Access

Supporting Information

for *Adv. Sci.*, DOI: 10.1002/advs.202104689

Achieving uniform Li plating/stripping at ultrahigh currents and capacities by optimising 3D nucleation sites and Li2Se-enriched SEI

Jiaqi Cao, Yonghui Xie, Yang Yang, Xinghui Wang, Wangyang Li, Qiaoli Zhang, Shun Ma, Shuying Cheng,* Bingan Lu**

Supporting Information

Achieving uniform Li plating/stripping at ultrahigh currents and capacities by optimising 3D nucleation sites and Li_2Se-enriched SEI

Experimental Section

Material Synthesis

Synthesis of Co-ZIF@CFC: Co-ZIF@CFC was synthesized via a solution method.[1] First, the commercial carbon fiber cloth (CFC, 4×4 cm^2) was treated in concentrated HNO_3 before use. Then, 1.1641g $Co(NO_3)_2·6H_2O$ (Aladdin) and 2.6272g 2-methylimidazol (2-MIM, Aladdin) were dissolved in 80 mL deionized water to form aqueous solutions, respectively. Subsequently, the 2-MIM solution was quickly poured into the $Co(NO_3)_2$ solution, and then the treated CFC (3 pieces) was immersed in the mixed solution for 5 hours. Finally, the Co-ZIF@CFC was taken out, rinsed with deionized water, and dried at 60 °C for further use.

Synthesis of $CoSe_2$-NC@CFC: The as-prepared Co-ZIF@CFC was annealed under Ar at 800 °C with a ramp rate of 5 °C min^{-1} for 2 h. Subsequently, the carbonized sample and excess Se powders were put into the middle and upstream of the tube furnace, respectively, and then annealed under N_2 at 450 °C with a ramp rate of 10 °C min^{-1} for 2 h. After cooling to room temperature, the $CoSe_2$-NC@CFC was obtained.

Preparation of Li/$CoSe_2$-NC@CFC anode: Li/$CoSe_2$-NC@CFC anode was obtained via an electroplating method. A $CoSe_2$-NC@CFC sample was used as a working electrode in a CR2025 coin cell, while Li foil was the counter/reference electrode (denoted as Li//$CoSe_2$-NC@CFC cell). The employed electrolyte was either-based electrolyte (1 M lithium bis(trifluoromethanesulfonyl)imide (LiTFSI) in 1,3-dioxolane

(DOL) and 1,2-dimethoxyethane (DME) (v/v=1:1) with 1% $LiNO_3$). The separator was Celgard 2400. The current density was set to 1 mA cm^{-2} during electrodeposition. Different Li deposition capacities (10 mAh cm^{-2} or 40 mAh cm^{-2}) can be achieved by setting the specific electroplating time. Finally, after disassembling the cell in the glove box and rinsed with DOL, the Li/$CoSe_2$-NC@CFC anode was obtained. In pouch cell test, the Li/$CoSe_2$-NC@CFC anode with 1.5×4 cm^2 was prepared via electroplating in ultrasealed electrolytic cell and performed on a Bio-Logic VSP workstation. The electroplating current density and time were set as 10 mA cm^{-2} and 1 h, respectively.

Material Characterization

The SEM and element analysis were explored via an FEI Helios G4 CX dual-beam field emission scanning electron microscope equipped with an energy dispersive X-ray spectrometer (EDX). To avoid air pollution of Li in SEM tests, the cycled cells were disassembled in the glovebox and rinsed with 1,3-dioxolane (DOL). After drying in the glovebox, the cycled electrodes were put into a sealed container filled with Ar and then transferred into the SEM chamber as quickly as possible. TEM, SAED, and element distributions were obtained by JEOL JEM-2100F field emission transmission electron microscope equipped with EDX. XRD characterizations were performed through a Rigaku SmartLab with Cu Kα radiation. Similarly, after rinsing by DOL and drying in the glovebox, the as-prepared Li/$CoSe_2$-NC@CFC anode was encapsulated in

the CR2032 Case (Kejing) with one side Kapton Window (10 mm) for further XRD analysis. XPS analysis was carried out by a Thermo ESCALAB 250 spectrometer. BET analysis was investigated by ASAP 2460 analyzer from Micromeritic.

Electrochemical Measurements

The batteries were assembled basing CR2025 coin cells in an Ar-filled glove box and measured via Neware battery cycler (CT-4008T, Shenzhen, China) at room temperature. The electrolyte was either-based electrolyte and the separator was Celgard 2400. To investigate the morphology evolutions of Li deposition on $CoSe_2$-NC@CFC or CFC, Li//$CoSe_2$-NC@CFC or Li//CFC cells were constructed and tested at 1 mA cm^{-2} with 10 mAh cm^{-2} or 40 mAh cm^{-2}. To evaluate Li stripping/plating behaviors, two identical Li/$CoSe_2$-NC@CFC anodes were assembled into symmetric cells. The capacity of Li pre-deposited in the $CoSe_2$-NC@CFC was 10 mAh cm^{-2} for the cycling capacity of 1 mAh cm^{-2} and 40 mAh cm^{-2} for the cycling capacity of 5, 10, and 20 mAh cm^{-2}. The electrochemical impedance spectroscopy (EIS) was conducted on a Bio-Logic VSP workstation with a frequency range from 100 kHz to 1 mHz. In full cell tests, the Li/$CoSe_2$-NC@CFC anode (10 mAh cm^{-2}) was paired with $LiFePO_4$ (LFP) cathode (active mass loading: ~6 mg cm^{-2}) to assemble the Li/$CoSe_2$-NC@CFC//LFP cell. The LFP cathode was prepared by a blade-casting method basing a mixed slurry, which is consisted of LFP, carbon

black, and polyvinylidene fluoride (8:1:1 wt%) in N-methyl-2-pyrrolidone (NMP). In pouch cell test, the active area of the LFP cathode was 1.5×3.5 cm^2 (active mass loading: ~10.97 mg).

Theoretical calculations

Theoretical calculations are performed using the Vienna Ab Initio Simulation Package (VASP), with the ionic potentials, including the effect of core electrons, being described by the projector augmented wave (PAW) method. In this work, the Perdew-Burke-Ernzerhof (PBE) GGA exchange-correlation functionals are used to relax the configurations of the Li^+ adsorbed on orthogonal $CoSe_2$ (111), cubic $CoSe_2$ (311) surface. A plane-wave energy cutoff of 500 eV is used in all calculations. All structures are geometrically relaxed until the total force on each ion is reduced below 0.02 eV $Å^{-1}$. The adsorption energy E_{ad} is defined as

$$E_{ad}=E_{adsorption}-E_{substrate}-E_{Li}$$

in which $E_{adsorption}$, $E_{substrate}$, and E_{Li} is the total energy of adsorbing Li on exposed surface, the total energy of substrate, and the total energy of Li, correspondingly. Similarly, the configurations and adsorption energies of Li at the sites of pyrrolic N, pridinic N, graphitic N, oxidized N, and C sites on the graphite sheet can be obtained accordingly.

Finite elemental analysis

The finite elemental analysis was executed using COMSOL Multiphysics software, in which the chosen physics module was secondary current distribution. The size of the two-dimensional model was set as 20×15 μm^2. The constructed nanostructures consist of vertical nanoflakes and embedded nanoparticles (corresponding to semicircles). The height and width of nanoflakes were set as 2 and 0.2 μm, respectively, while the diameter of nanoparticles was 100 nm. The gaps of adjacent nanoflakes and top-bottom nanoparticles were set as 1.5 and 0.2 μm, respectively. The whole simulated area was full of the bulk electrolyte (1 M LiTFSI in DOL/DME (v/v=1:1)) with a conductivity of 1.1 S m^{-1}.[2] The performed current density was 10 mA cm^{-2} and the dynamic expression type was Butler Volmer. The simulated process was carried out in steady-state at room temperature.[3]

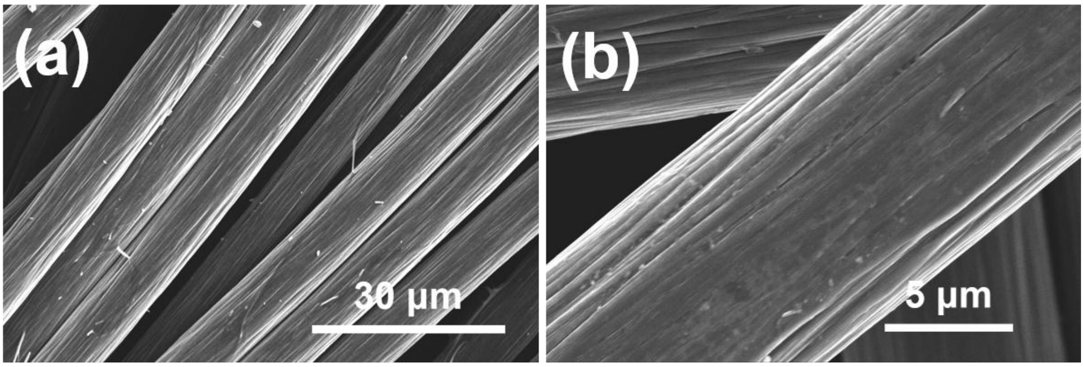

Figure S1. Low (a) and high (b) magnification SEM images of the CFC.

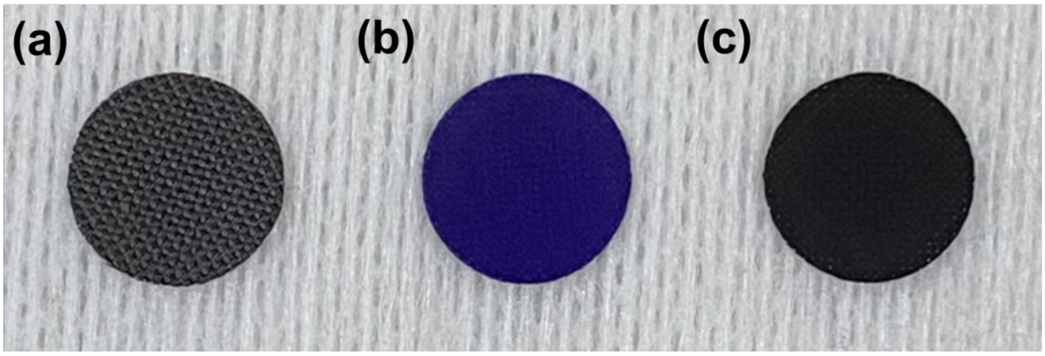

Figure S2. The optical photos of (a) CFC, (b) Co-ZIF@CFC, and (c) $CoSe_2$-NC@CFC.

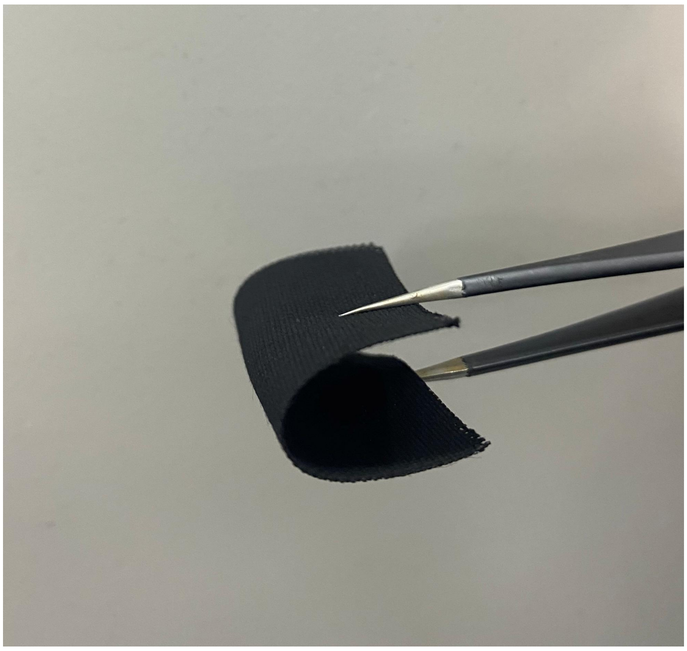

Figure S3. The flexibility illustration of $CoSe_2$-NC@CFC.

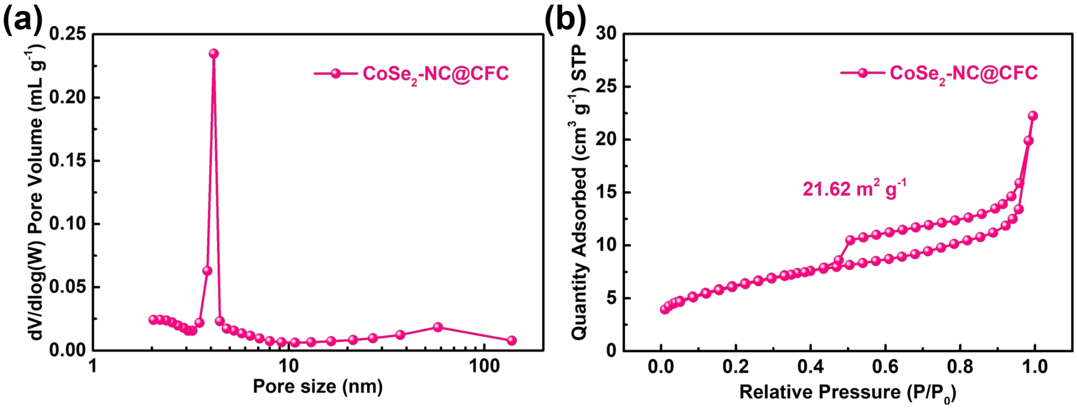

Figure S4. (a) BET analysis and (b) N_2 adsorption-desorption isothermal curves of $CoSe_2$-NC@CFC.

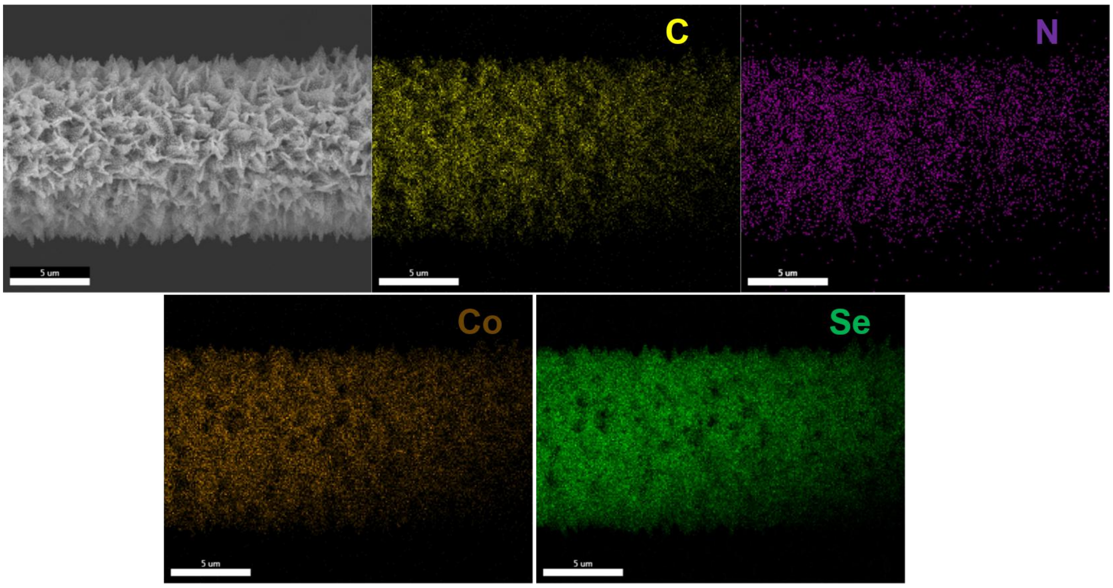

Figure S5. The SEM and corresponding elemental mapping images of $CoSe_2$-NC@CFC.

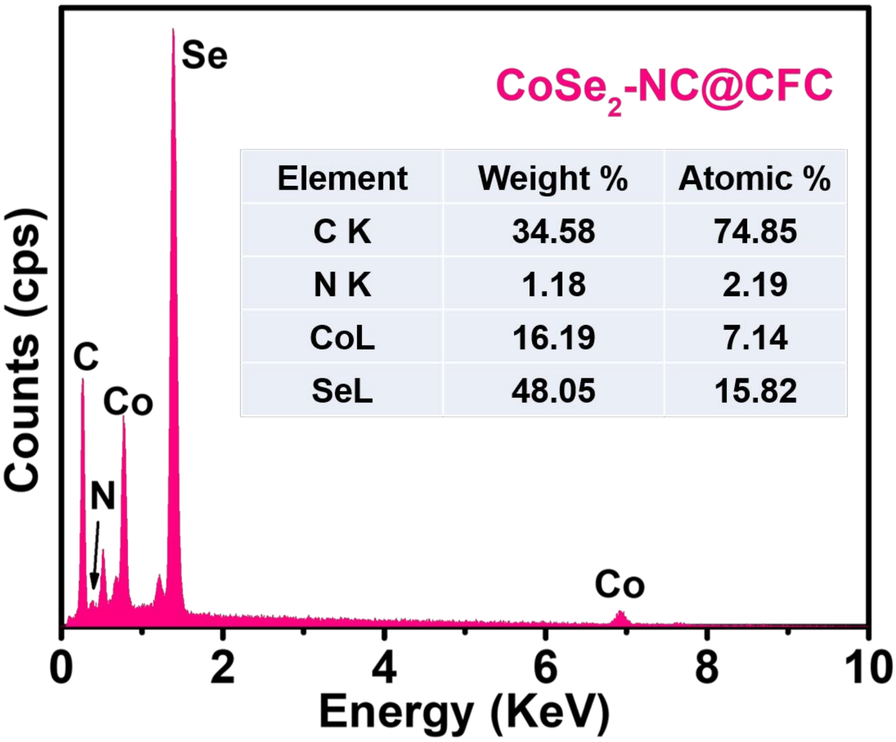

Figure S6. The EDX analysis of the $CoSe_2$-NC@CFC.

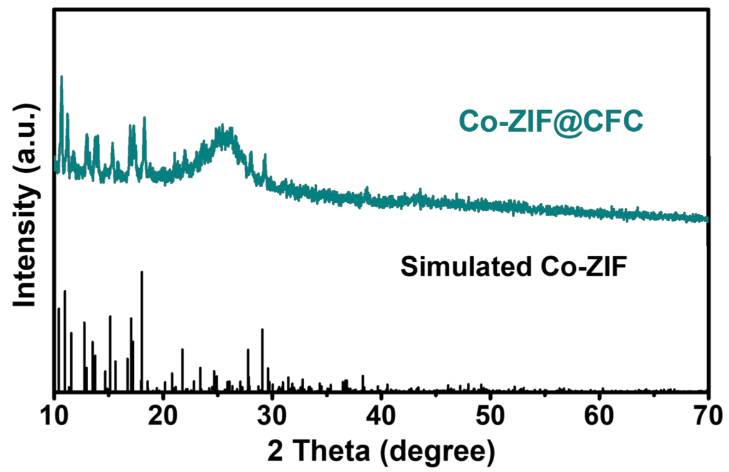

Figure S7. XRD pattern of Co-ZIF@CFC.

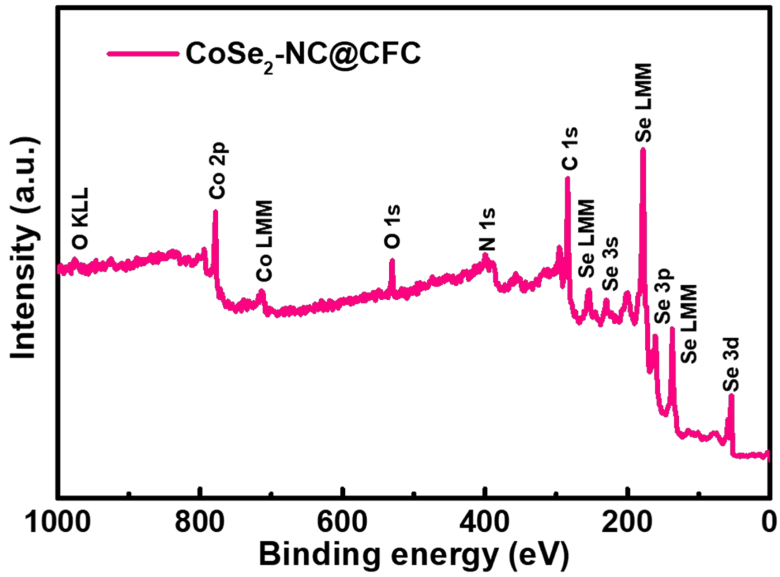

Figure S8. The full XPS survey of $CoSe_2$-NC@CFC.

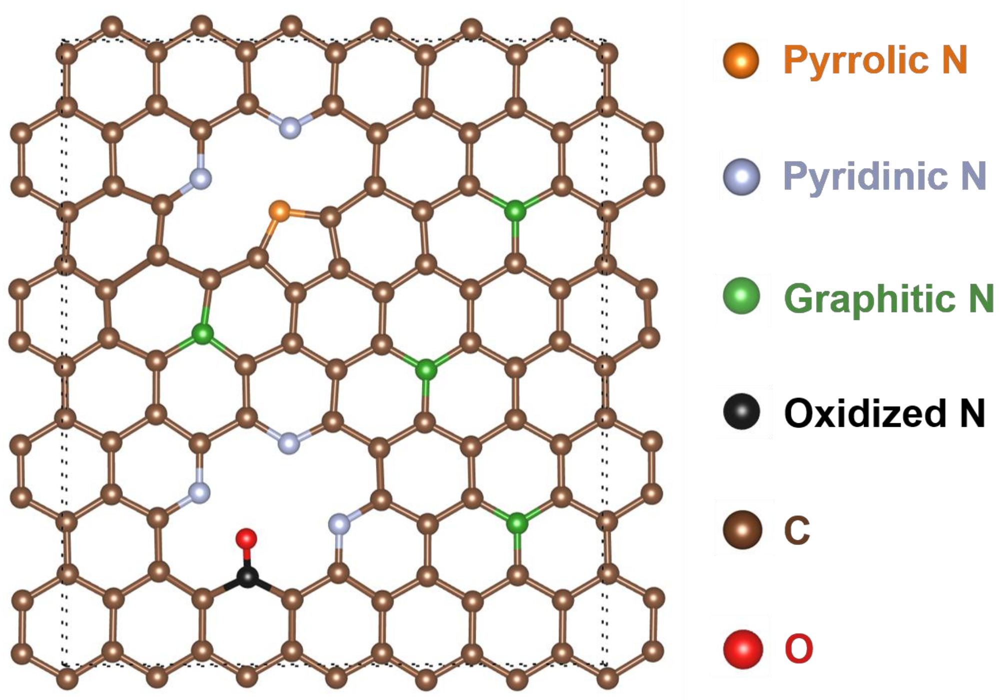

Figure S9. The schematic diagram of N configuration in the $CoSe_2$-NC@CFC.

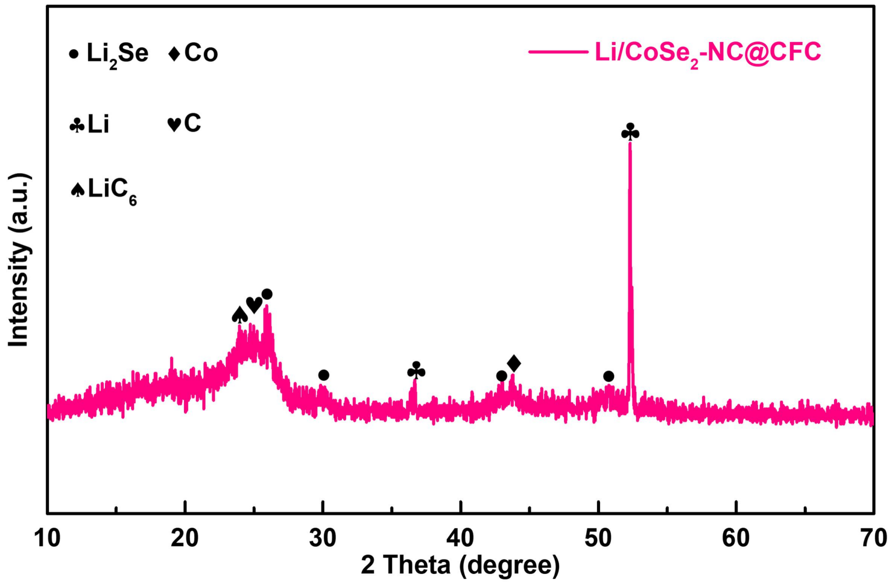

Figure S10. The XRD pattern of $CoSe_2$-NC@CFC after electroplating 10 mAh cm^{-2} Li at 1 mA cm^{-2}.

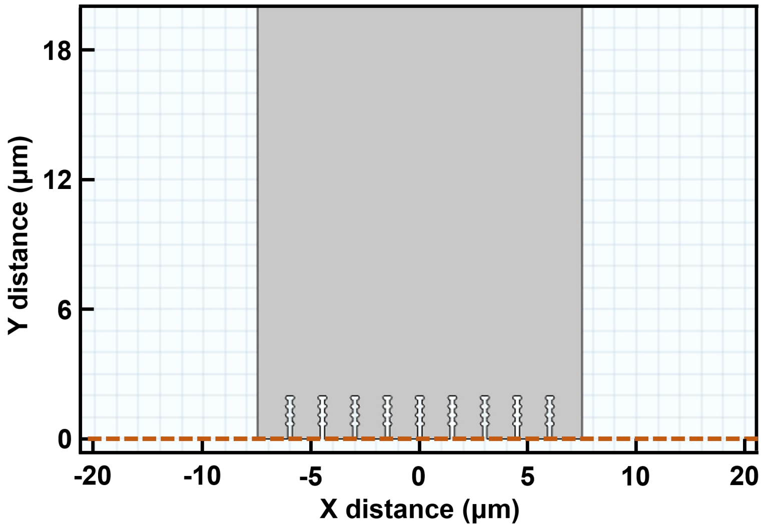

Figure S11. The constructed module of $CoSe_2$-NC nanoflake arrays.

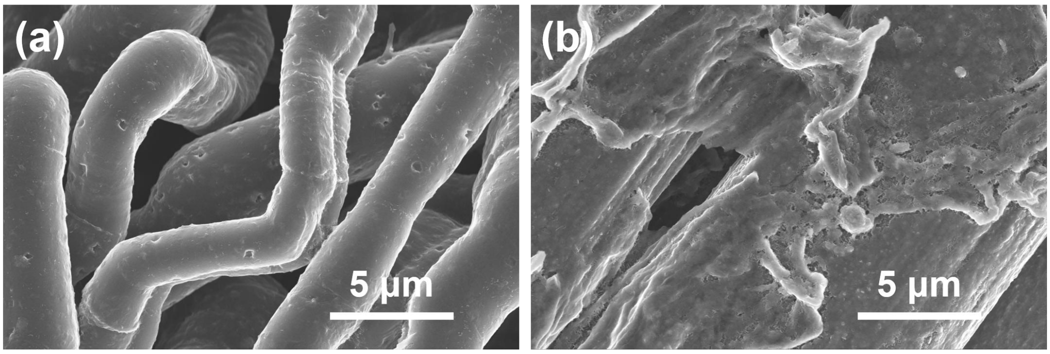

Figure S12. The SEM images of CFC (a) after depositing 10 mAh cm^{-2} Li and then (b) charging to 1 V.

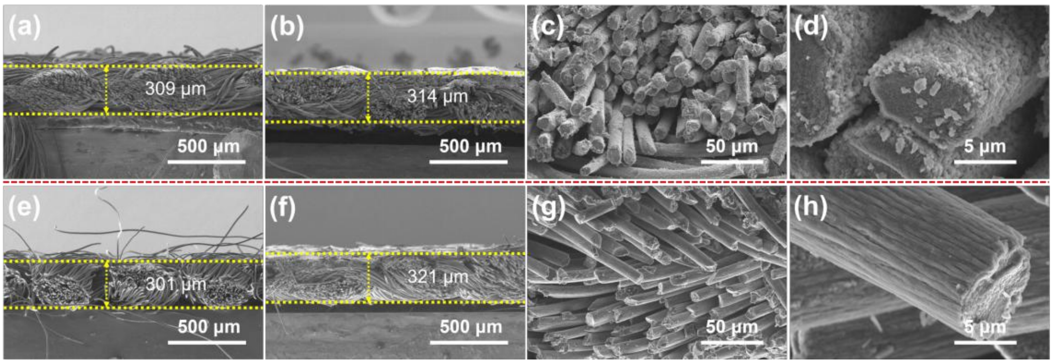

Figure S13. The cross-section SEM images of pristine $CoSe_2$-NC@CFC (a) before and (b-d) after plating 10 mAh cm^{-2} Li. The cross-section SEM images of pristine CFC (a) before and (b-d) after plating 10 mAh cm^{-2} Li.

The cross-section SEM images in Figure S13 compare the volume changes of $CoSe_2$-NC@CFC and CFC before and after 10 mAh cm^{-2} Li plating. As shown in Figure S13a-b and e-f, the thickness of Li/$CoSe_2$-NC@CFC is ~314 μm with a negligible volume change of 1.6% (~309 μm) after 10 mAh cm^{-2} Li plating, while the Li/CFC increases from ~301 to ~321 μm (6.6%), suggesting that $CoSe_2$-NC@CFC could store more Li inside the electrode. From the magnified SEM images shown in Figure S13c-d and g-h, it can be clearly observed that the internal $CoSe_2$-NC nanoflake arrays are covered with metallic Li, while negligible Li is deposited on internal fibers of CFC.

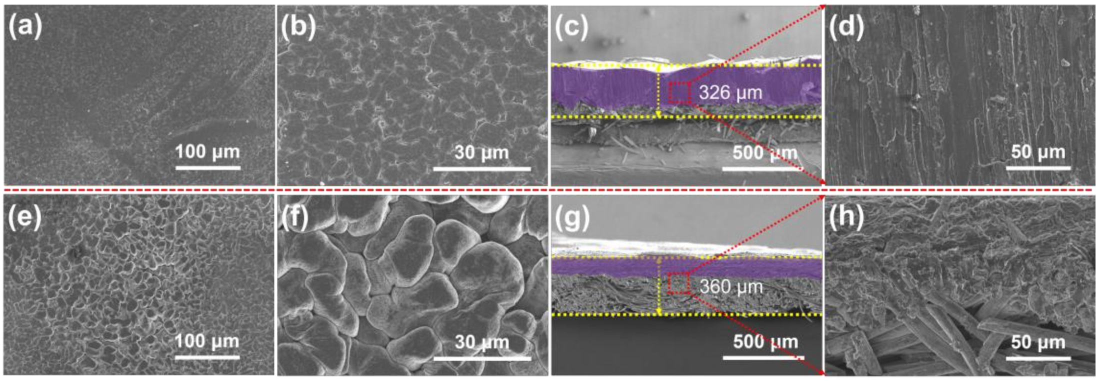

Figure S14. The (a-b) top-view and (c-d) cross-section SEM images of $CoSe_2$-NC@CFC after plating 40 mAh cm^{-2} Li. The (e-f) top-view and (g-h) cross-section SEM images of CFC after plating 40 mAh cm^{-2} Li. The purple parts represent the regions of Li deposition.

Compared with flat morphology and steady volume of $CoSe_2$-NC@CFC after plating 40 mAh cm^{-2} Li (Figure S14a-d), the surface of CFC exists numerous Li dendrites, which is detrimental to long-term cycling stability (Figure S14e-f). More importantly, the Li deposition cannot be completely confined within the CFC framework, resulting in the huge volume change of CFC from ~301 μm to ~360 μm (Figure S14g-h).

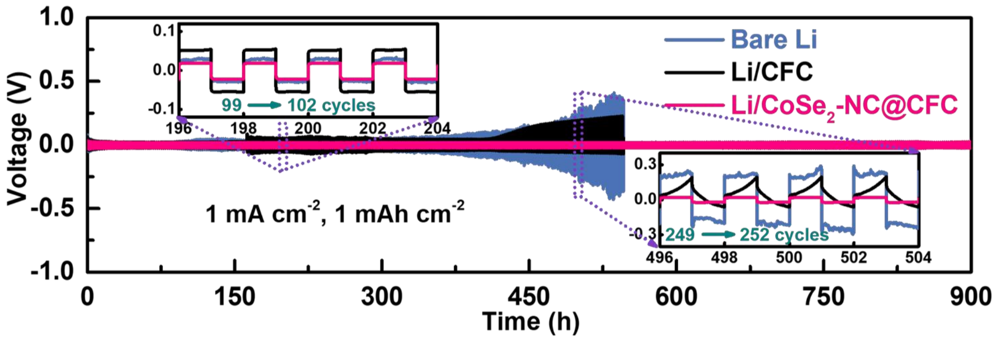

Figure S15. Galvanostatic cycling of bare Li, Li/CFC, and Li/$CoSe_2$-NC@CFC with a capacity of 1 mAh cm^{-2} at 1 mA cm^{-2}.

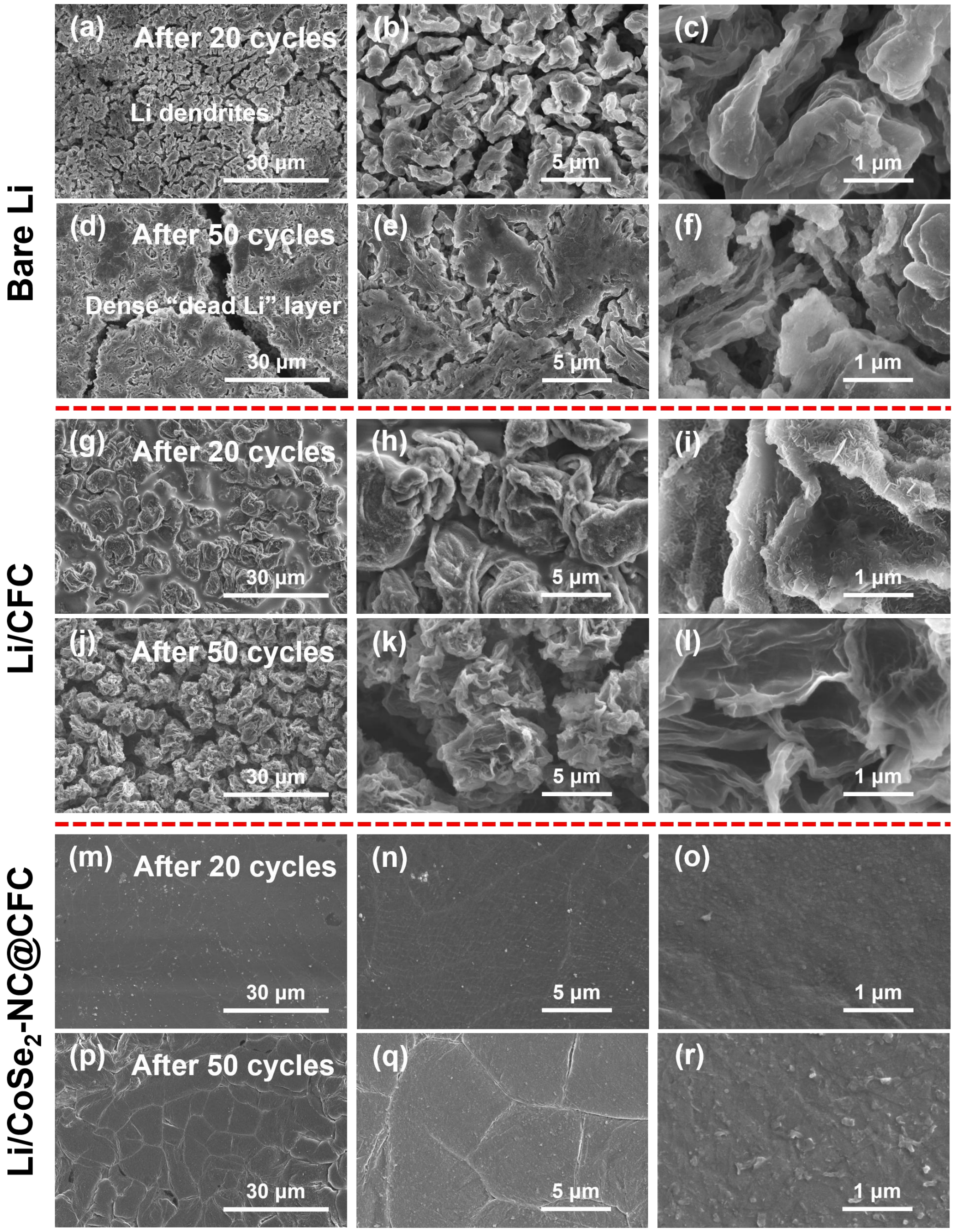

Figure S16. The SEM images of morphologies of bare Li, Li/CFC, and Li/$CoSe_2$-NC@CFC anodes after 20 and 50 cycles at 1 mA cm^{-2} with 1 mAh cm^{-2}. Bare Li after (a-c) 20 and (d-f) 50 cycles. Li/CFC after (g-i) 20 and (j-l) 50 cycles. Li/$CoSe_2$-NC@CFC after (m-o) 20 and (p-r) 50 cycles.

As shown in Figure S16a-l, after different cycles, the morphologies of bare Li and Li/CFC all engender the growth of Li dendrites. As the cycle continues, partial Li

dendrites will transform into "dead Li" and hinder the transport of Li^+ during the striping/plating process, resulting in the increase of internal resistance and overpotential. By comparison, Li/$CoSe_2$-NC@CFC anodes maintain flat and dense morphology no matter after 20 cycles or 50cycles, which is beneficial to protect SEI integrity and suppress the formation of "dead Li", possessing the superior cycling performance (Figure S16m-r).

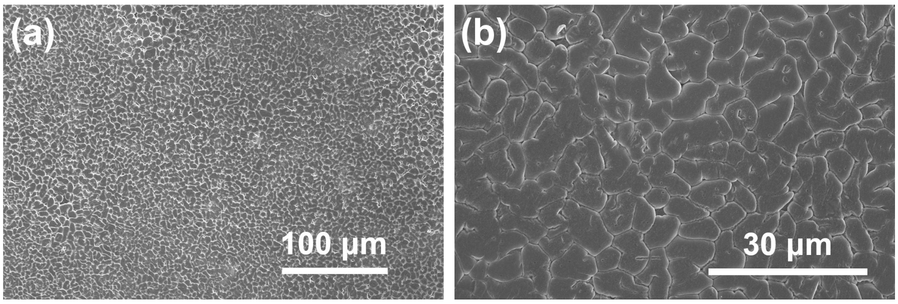

Figure S17. The top-view SEM images of Li/$CoSe_2$-NC@CFC anode after 20 cycles with 10 mAh cm^{-2} at 10 mA cm^{-2}.

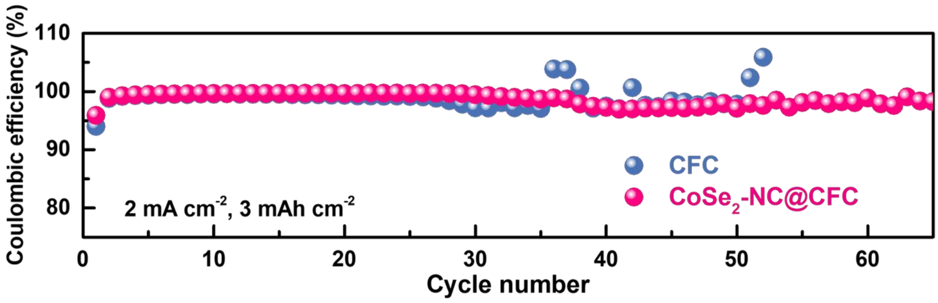

Figure S18. The Coulombic efficiency comparison of the $CoSe_2$-NC@CFC//Li and CFC//Li half cells at 2 mA cm^{-2} with a capacity of 3 mAh cm^{-2}.

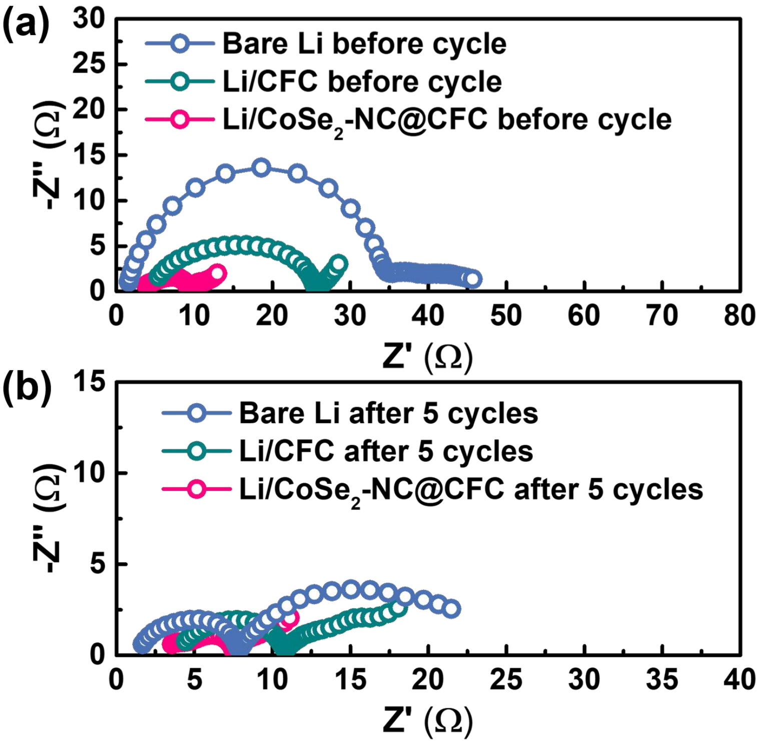

Figure S19. Nyquist plots in symmetric cells with bare Li, Li/CFC and Li/$CoSe_2$-NC@CFC anodes (a) before and (b) after 5 cycles at 1.0 mA cm^{-2}.

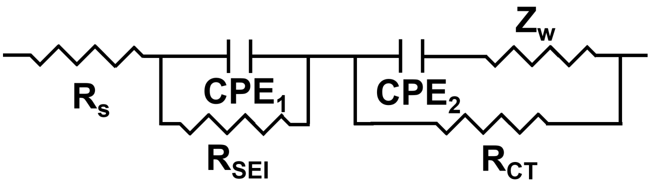

Figure S20. The equivalent circuit for Figure S19.

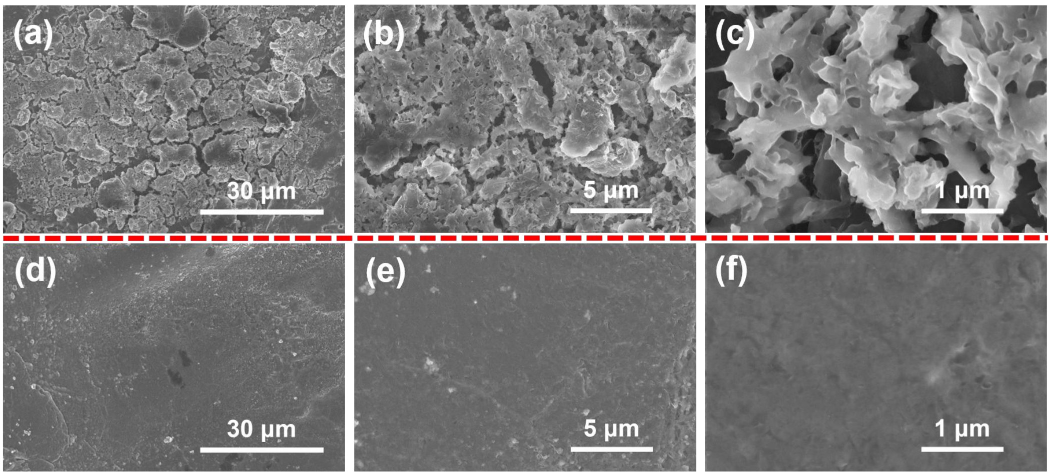

Figure S21 The top-view SEM images of (a-c) bare Li//LFP and (d-f) Li/$CoSe_2$-NC@CFC//LFP after rate test.

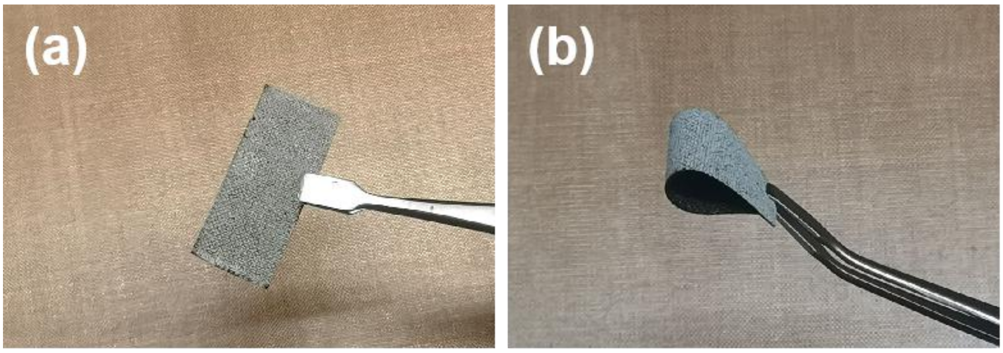

Figure S22. (a) Optical photo of Li/$CoSe_2$-NC@CFC after electroplating process. (b) The illustration for flexibility of Li/$CoSe_2$-NC@CFC.

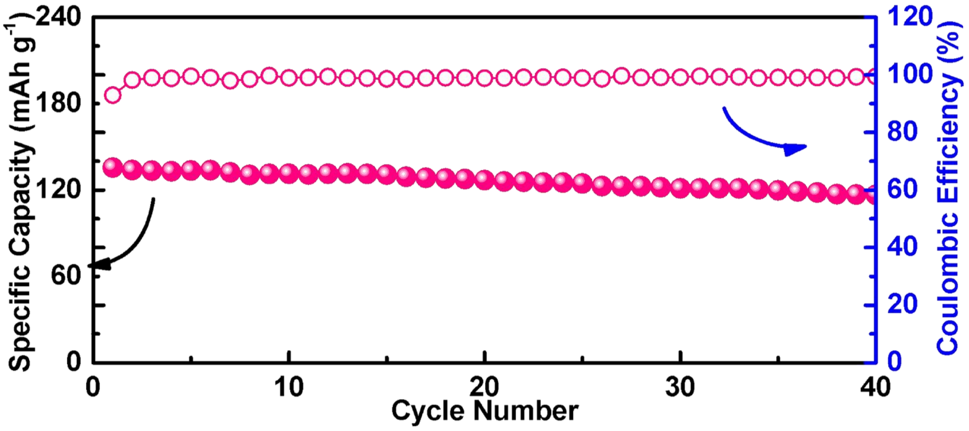

Figure S23. The cycling performance of Li/$CoSe_2$-NC@CFC//LFP flexible pouch cell under flat state at 0.5 C.

Table S1. Comparison of performances of symmetrical cells with the Li/$CoSe_2$-NC@CFC anode with various previously reported Li metal anodes under high current density or high cycling capacity.

Electrode	Current density (mA cm^{-2})/ Areal capacity (mAh cm^{-2})	Cycle time (h)	Cycle number
This work	5/5	2000	1000
	10/5	1000	1000
	10/10	1600	800
	5/20	1600	200
	10/20	280	70
NF/NCNT/Li[4]	3/3	400	200
CFC/Co-NC@Li[5]	5/10	200	50
CoSe@C/Li[6]	10/10	1000	500
Li/Li_xLM_y[7]	1/8	600	~38
SEI@Li/SN[8]	5/10	1500	375
CC@CN-Co@Li[9]	5/5	1000	500
Cu@MC@Li[10]	3/1	150	225
CNF@Ni-Li[11]	1/5	1000	100
Cu/Li[12]	1/6	600	50

Table S2 Fitted impedance results of Figure S19.

	Bare Li			Li/CFC			Li/Cu-NC@CFC//LFP		
	R_s (Ω)	R_{SEI} (Ω)	R_{CT} (Ω)	R_s (Ω)	R_{SEI} (Ω)	R_{CT} (Ω)	R_s (Ω)	R_{SEI} (Ω)	R_{CT} (Ω)
**pristine**	1.4	4.5	27.1	4.2	12.1	9.4	3.2	3.1	2.9
**5st**	1.4	3.3	2.8	4.0	3.5	3.0	2.8	2.4	2.3

Reference

[1] Z. Wang, J. Shen, J. Liu, X. Xu, Z. Liu, R. Hu, L. Yang, Y. Feng, J. Liu, Z. Shi, Self-supported and flexible sulfur cathode enabled via synergistic confinement for high-energy-density lithium–sulfur batteries, Adv. Mater. 31 (2019) 1902228.

[2] B. Liu, Y. Zhang, G. Pan, C. Ai, S. Deng, S. Liu, Q. Liu, X. Wang, X. Xia, J. Tu, Ordered lithiophilic sites to regulate Li plating/stripping behavior for superior lithium metal anodes, J. Mater. Chem. A 7 (2019) 21794-21801.

[3] S. Li, Q. Liu, J. Zhou, T. Pan, L. Gao, W. Zhang, L. Fan, Y. Lu, Hierarchical Co3O4 Nanofiber–Carbon Sheet Skeleton with Superior Na/Li-Philic Property Enabling Highly Stable Alkali Metal Batteries, Adv. Funct. Mater. 29 (2019) 1808847.

[4] Z. Zhang, J. Wang, X. Yan, S. Zhang, W. Yang, Z. Zhuang, W.-Q. Han, In-situ growth of hierarchical N-doped CNTs/Ni Foam scaffold for dendrite-free lithium metal anode, Energy Storage Mater. 29 (2020) 332-340.

[5] G. Jiang, N. Jiang, N. Zheng, X. Chen, J. Mao, G. Ding, Y. Li, F. Sun, Y. Li, MOF-derived porous Co3O4-NC nanoflake arrays on carbon fiber cloth as stable hosts for dendrite-free Li metal anodes, Energy Storage Mater. 23 (2019) 181-189.

[6] J. He, A. Manthiram, 3D CoSe@ C Aerogel as a Host for Dendrite-Free Lithium-Metal Anode and Efficient Sulfur Cathode in Li–S Full Cells, Adv. Energy Mater. 10 (2020) 2002654.

[7] B. Han, D. Xu, S.S. Chi, D. He, Z. Zhang, L. Du, M. Gu, C. Wang, H. Meng, K. Xu, 500 Wh kg− 1 Class Li Metal Battery Enabled by a Self-Organized Core–Shell Composite Anode, Adv. Mater. 32 (2020) 2004793.

[8] B. Liu, Y. Zhang, Z. Wang, C. Ai, S. Liu, P. Liu, Y. Zhong, S. Lin, S. Deng, Q. Liu, Coupling a Sponge Metal Fibers Skeleton with In Situ Surface Engineering to Achieve Advanced Electrodes for Flexible Lithium–Sulfur Batteries, Adv. Mater. 32 (2020) 2003657.

[9] T. Zhou, J. Shen, Z. Wang, J. Liu, R. Hu, L. Ouyang, Y. Feng, H. Liu, Y. Yu, M. Zhu, Regulating Lithium Nucleation and Deposition via MOF-Derived Co@C-Modified Carbon Cloth for Stable Li Metal Anode, Adv. Funct. Mater. 30

(2020) 1909159.

[10] J. Qian, S. Wang, Y. Li, M. Zhang, F. Wang, Y. Zhao, Q. Sun, L. Li, F. Wu, R. Chen, Lithium Induced Nano-Sized Copper with Exposed Lithiophilic Surfaces to Achieve Dense Lithium Deposition for Lithium Metal Anode, Adv. Funct. Mater. 31 (2021) 2006950.

[11] Z. Zeng, W. Li, X. Chen, X. Liu, Bifunctional 3D Hierarchical Hairy Foam toward Ultrastable Lithium/Sulfur Electrochemistry, Adv. Funct. Mater. 30 (2020) 2004650.

[12] S. Huang, L. Chen, T. Wang, J. Hu, Q. Zhang, H. Zhang, C. Nan, L.-Z. Fan, Self-Propagating Enabling High Lithium Metal Utilization Ratio Composite Anodes for Lithium Metal Batteries, Nano Lett. 21 (2021) 791-797.
